# Factors associated with retention in Option B+ in Malawi: a case control study

**DOI:** 10.7448/IAS.20.01.21464

**Published:** 2017-04-27

**Authors:** Risa M Hoffman, Khumbo Phiri, Julie Parent, Jonathan Grotts, David Elashoff, Paul Kawale, Sara Yeatman, Judith S Currier, Alan Schooley

**Affiliations:** ^a^ Department of Medicine, Division of Infectious Diseases, David Geffen School of Medicine, University of California, Los Angeles, Los Angeles, CA, USA; ^b^ Partners in Hope Medical Center, Lilongwe, Malawi; ^c^ Department of Medicine Statistics Core, David Geffen School of Medicine, University of California, Los Angeles, Los Angeles, CA, USA; ^d^ Department of Public Health, Nkhoma Hospital, Nkhoma, Malawi; ^e^ Malawi and eHealth Research Group, Global Health Academy, Usher Institute of Population Health Sciences and Informatics, The University of Edinburgh, Edinburgh, UK; ^f^ Department of Health and Behavioral Sciences, University of Colorado, Denver, Denver, CO, USA

**Keywords:** HIV/AIDS, Option B+, Malawi, retention, prevention of mother-to-child transmission

## Abstract

**Introduction**: There are limited data on factors associated with retention in Option B+. We sought to explore the characteristics of women retained in Option B+ in Malawi, with a focus on the role of HIV disclosure, awareness of partner HIV status, and knowledge around the importance of Option B+ for maternal–child health.

**Methods**: We performed a case-control study of HIV-infected women in Malawi initiated on antiretroviral therapy (ART) under Option B+. Cases were enrolled if they met criteria for default from Option B+ (out of ART for >60 days), and controls were enrolled in approximately 3:1 ratio if they were retained in care for at least 12 months. We surveyed socio-demographic characteristics, HIV disclosure and awareness of partner HIV status, self-report about receiving pre-ART education, and knowledge of Option B+. Univariate logistic regression was performed to determine factors associated with retention. Multivariate logistic regression model was used to evaluate the relationship between HIV disclosure, Option B+ knowledge, and retention after adjusting for age, schooling, and travel time to clinic.

**Results**: We enrolled 50 cases and 153 controls. Median age was 30 years (interquartile range (IQR) 25–34), and the majority (82%) initiated ART during pregnancy at a median gestational age of 24 weeks (IQR 16–28). Ninety-one per cent of the cases (39/43) who started ART during pregnancy defaulted by three months postpartum. HIV disclosure to the primary sex partner was more common among women retained in care (100% versus 78%, *p* < 0.001). Odds of retention were significantly higher among women with: age >25 years (odds ratio (OR) 2.44), completion of primary school (OR 3.06), awareness of partner HIV status (OR 5.20), pre-ART education (OR 6.17), higher number of correct answers to Option B+ knowledge questions (OR 1.82), and support while taking ART (OR 3.65). Pre-ART education and knowledge were significantly correlated (*r* = 0.43, *p* < 0.001). In multivariate analysis, awareness of partner HIV status (OR 4.07, 95% confidence interval (CI) 1.51–10.94, *p* = 0.02) and Option B+ knowledge (OR 1.60, 95% CI 1.15–2.23, *p* = 0.004) remained associated with retention.

**Conclusions**: Interventions that address partner disclosure and strengthen pre-ART education around the benefits of ART for maternal and child health should be evaluated to improve retention in Malawi’s Option B+ programme.

## Introduction

Malawi was among the first countries in Africa to implement the Option B+ prevention of mother-to-child transmission (PMTCT) of HIV policy in July 2011 [1]. Under this policy, all HIV-infected pregnant women and breastfeeding women are started on antiretroviral therapy (ART) with a three-drug regimen (efavirenz, tenofovir, and lamivudine) and continued on treatment for life. Initiation is done regardless of CD4 count and clinical disease stage. The programme has achieved high rates of coverage, with almost 80% of pregnant women on ART through decentralization of service delivery utilizing antenatal clinics (ANCs) and maternity clinics as sites for initiation [2]. Option B+ has reduced rates of mother-to-child HIV transmission and resulted in improvements in rates of antepartum ART initiation, infant testing rates, and ART initiation in HIV-infected infants [2–5].

Despite these successes, there are high rates of loss to follow-up from Option B+, particularly early after ART initiation, with recent Ministry of Health quarterly report data showing Option B+ retention of 76.8% at 12 months after ART initiation. Retention further declines 24 and 36 months after starting ART (70.8% and 69.7%, respectively), and rates have not changed significantly as the Option B+ programme has matured [6]. Factors associated with loss to follow-up include receiving an HIV diagnosis on the same day as ART initiation [7,8], younger age [7,9], cost and time to travel to clinic [9], low knowledge around the benefits of Option B+ for maternal and infant health [9], and non-disclosure of HIV status [10,11]. The majority of studies reporting these data are from women who missed appointments and/or defaulted and did not allow for comparison to women retained in care.

Early loss to follow-up from Option B+ is a threat to achieving maximal benefits. Further work is needed to help women remain in care and on ART during pregnancy, breastfeeding, and future subsequent pregnancies. We sought to explore characteristics of women retained in Option B+ in central Malawi using a case-control design, with a focus on identifying modifiable factors that could be targets for programme interventions to improve retention in care. We hypothesized that disclosure of HIV status to the primary sex partner, awareness of partner HIV status, and higher knowledge about the importance of Option B+ for maternal and child health would be associated with retention in care after adjustment for the established factors of age, education, and distance from the ART clinic.

## Methods

This study was performed under the umbrella of Expanding QUality ImProvement (EQUIP)-Malawi, a President’s Emergency Plan for AIDS Relief-United States Agency for International Development-funded HIV care and treatment programme. Women were recruited from 14 EQUIP-supported ART clinics in central Malawi between October 2014 and April 2015. Central Malawi contains 9 of the country’s 27 districts and is largely rural with the exception of the capital of Lilongwe. Women were recruited from rural sites in the central region. Controls were defined as HIV-infected women 18 years of age and older who started ART under Option B+ and were retained in care ≥12 months. Women in the control arm were approached during routine ART clinic visits. Cases were defined as women who met criteria for default from Option B+ based on the Malawi national programme definition (out of ART for >60 days). Of a total of 289 women who had defaulted from sites, 50 (17%) were located through standard tracing EQUIP-supported tracing protocols. At the time of tracing, women were asked if they were interested in participating in the study and were given the option to complete the survey in a private place in their community or to complete the survey in clinic at the time of their return appointment. All 50 women approached for the study consented to participate. Controls were enrolled in a 3:1 ratio. Written informed consent was obtained from all women.

All women completed a 1-hour survey, which was administered by a Malawian research assistant who asked questions in the local language (Chichewa). Survey domains included socio-demographics including time for travel to ART clinic, HIV diagnosis and treatment characteristics, and disclosure status (disclosure to partner and awareness of partner HIV status). Women were asked about the specific types of education they received prior to starting ART (pre-ART education) and were asked to answer six multiple-choice questions assessing knowledge about Option B+, as follows: (1) Does ART help when you are pregnant to protect the baby from HIV? (2) Does ART help when you are breastfeeding to protect the baby from HIV? (3) How long is ART needed for women who start treatment during pregnancy or breastfeeding? (4) When should a baby born to an HIV-positive woman get his/her first test for HIV? (5) Does ART help to prevent HIV-negative partners from getting infected through sex? (6) Is it safe to become pregnant while taking ART? Finally, a list of barriers to retention in care was compiled based on a literature review, and all women were asked to select any barriers from the list influencing their ability to remain in care. A similar process was utilized to identify facilitators to ART.

Approval for research was granted by the Malawi College of Medicine Research and Ethics Committee in Blantyre (P.07/14/1603) and by the University of California, Los Angeles Institutional Research Board (IRB #14–000287).

### Statistical methods

Data were entered into a Microsoft Access database and transferred into R Statistical Computing Environment (version 3.3, Vienna, Austria) for analysis. Summary statistics were performed on basic socio-demographic and HIV diagnosis and treatment characteristics. Univariate logistic regression was performed to determine factors associated with retention. A multiple logistic regression model was used to evaluate HIV disclosure, awareness of partner HIV status, and Option B+ knowledge with retention in care, while adjusting for age, education, and travel time to clinic. Factors selected for adjustment in the multivariate model were based on published literature showing these variables to be independently associated with retention among pregnant and postpartum women. The covariance matrix of each multiple regression model was checked and there were no correlations between variables greater than 0.4. The variance inflation factor was also checked for each model, and no variable had a factor greater than 1.5. A second analysis was performed comparing barriers to retention in care reported by cases and controls using chi-square and Fisher’s exact tests. Summary statistics were used to characterize facilitators of retention in Option B+.

## Results

We enrolled 50 cases and 153 controls from October 2014 to April 2015. Median age was 30 years (interquartile range (IQR) 25–34) and 115 (57%) were married/monogamous, 30 (15%) married/polygamous, and 58 (29%) were not in a current partnership. The majority of women (*N* = 175, 86%) were diagnosed with HIV during their most recent pregnancy, and of these, 171 (98%) were initiated on ART the same day they were tested for HIV. Of the women known to be HIV infected prior to the current pregnancy, 16 (57%) reported taking short-course antiretrovirals for PMTCT at least once. The median gestational age at ART initiation was 24 weeks (IQR 16–28). Among defaulters (cases), 20 (40%) were lost to follow-up prior to delivery, and 18 (36%) were lost between delivery and three months postpartum. The median weeks from ART initiation to default was 8.6 (IQR 0–26 and range 0–145). Among cases lost to follow-up by three months postpartum, five either declined to initiate ART or received a bottle of ART but never started.

All women retained in care who reported a primary sex partner had disclosed their HIV status to this individual, compared to 78% of women who defaulted (*p* < 0.001). Odds of retention were significantly higher among women with age >25 years (odds ratio (OR) 2.44), completion of primary school (OR 3.06), awareness of partner HIV status (OR 5.20), those who received pre-ART education (OR 6.17), those with a higher number of correct answers to Option B+ knowledge questions (OR 1.82), and individuals with one or more methods of support while taking ART (OR 3.65). Travel time of >3 hours to clinic and later gestational age at ART initiation were associated with significantly reduced odds of retention (OR 0.13 and 0.95, respectively) (Table 1).

**Table 1.** Univariate regression for the outcome of retention in Option B+, *n* = 203^a^.

Variable^a^	Cases^c^ (*n* = 50)	Controls^c^ (*n* = 153)	Odds ratio (95% confidence interval)	*p*-Value^b^
Age				
18–25	21 (43)	36 (24)	1.00 (Reference)	0.01
>25	28 (57)	117 (76)	2.44 (1.24–4.81)	
Education				
Less than primary school	44 (88)	108 (71)	1.00 (Reference)	0.009
Primary school and beyond	6 (12)	45 (29)	3.06 (1.22–7.69)	
Duration to travel to ART clinic				
≤1 hour	12 (24)	82 (54)	1.00 (Reference)	<0.001
>1 hour up to 3 hours	27 (54)	61 (40)	0.33 (0.15–0.70)	
>3 hours	11 (22)	10 (6)	0.13 (0.05–0.37)	
Diagnosed with HIV during the most recent pregnancy?				
No	4 (8)	24 (16)	1.00 (Reference)	0.15
Yes	46 (92)	129 (84)	0.47 (0.15–1.43)	
ART initiation on the same day as HIV diagnosis?				
No	2 (4)	13 (9)	1.00 (Reference)	0.26
Yes	44 (96)	127 (91)	0.44 (0.10–2.03)	
Status at ART initiation				
Pregnancy	43 (86)	123 (80)	1.00 (Reference)	0.36
Breastfeeding	7 (14)	30 (20)	1.50 (0.61–3.66)	
Gestational age in weeks at ART start among those pregnant, median (IQR)	26 (16–32)	24 (16–28)	0.95 (0.91–0.99)	0.01
Participant aware of partner’s HIV status?				
No	17 (47)	16 (15)	1.00 (Reference)	0.001
Yes	19 (53)	93 (85)	5.20 (2.24–12.07)	
Not applicable	14(28)	44 (28.8)	3.34 (1.34–8.30)	
Received pre-ART education^d^				
No	28 (56)	26 (17)	1.00 (Reference)	<0.001
Yes	22 (44)	126 (83)	6.17 (3.06–12.43)	
Correctly answered Option B+ knowledge questions (out of six questions), median (IQR)				
Number of knowledge questions answered correctly	4 (3–4)	4 (4–5)	1.82 (1.35–2.45)	<0.001
0–2	11 (22)	12 (8)	1.00 (Reference)	0.006
3–4	30 (60)	86 (56)	2.63 (1.05–6.58)	
5–6	9 (18)	55 (36)	5.60 (1.90–16.49)	
				
No	28 (56)	26 (17)	1.00 (Reference)	<0.001
Yes	22 (44)	126 (83)	6.17 (3.06–12.43)	
Received support while taking ART^e^				
No	6 (13)	6 (4)	1.00 (Reference)	0.04
Yes	40 (87)	146 (96)	3.65 (1.12–11.93)	

**^a^**All variables reported as *N* (%) unless otherwise noted. **^b^**Calculated with chi-square test. ^c^Cases defined by Malawi National definition of default = out of ART for more than 60 days; controls were required to be in care for at least 12 months. ^d^Based on self-report of learning about five different Option B+ topics prior to starting ART. ^e^Support was defined by use of support groups at the clinic or in the community, reminders for appointments, peer mentoring, or other counselling. ART: antiretroviral therapy; IQR: interquartile range.

In the multivariate model, pre-ART education and knowledge were significantly correlated (*r* = 0.43, *p* < 0.001), and therefore, only knowledge was included in the analysis. Additionally, disclosure to partner was excluded from multivariate analysis because 100% of controls disclosed. Awareness of partner HIV status (OR 4.07, 95% confidence interval (CI) 1.51–10.94, *p* = 0.02) and Option B+ knowledge (OR 1.60, 95% CI 1.15–2.23, *p* = 0.004) remained associated with retention (Table 2).

**Table 2.** Multivariate regression for the outcome of retention in Option B+, *n* = 203.

Variable^a^	Cases *N* = 50	Controls *N* = 153	Multivariate odds ratio (95% confidence interval)	*p*-Value
Age				
18–25	21 (43)	36 (24)	1.00 (Reference)	0.015
>25	28 (57)	117 (76)	2.71 (1.21–6.06)	
Education				
Less than primary school	44 (88)	108 (71)	1.00 (Reference)	0.109
Primary school and beyond	6 (12)	45 (29)	2.24 (0.80–6.26)	
Duration of travel to antiretroviral therapy clinic				
≤1 hour	12 (24)	82 (54)	1.00 (Reference)	0.005
>1–3 hours	27 (54)	61 (40)	0.39 (0.17–0.90)	
≥3 hours	11 (22)	10 (6)	0.16 (0.05–0.53)	
Participant aware of partner’s HIV status?				
No	17 (47)	16 (15)	1.00 (Reference)	0.016
Yes	19 (53)	93 (85)	4.07 (1.51–10.94)	
No partner	14(28)	44 (29)	3.70 (1.25–10.96)	
Number of knowledge questions answered correctly	4 (3–4)^b^	4 (4–5)^b^	1.60 (1.15–2.23)	0.004

^a^All variables reported as *N* (%) unless otherwise noted. ^b^Data presented as median (interquartile range).

**Figure 1.**
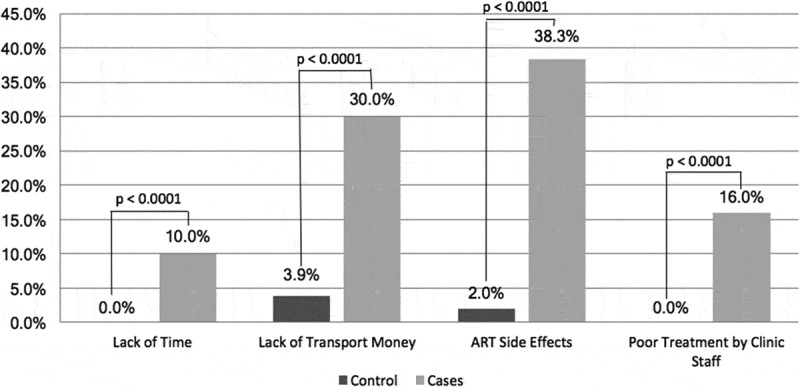
Common barriers to retention in Option B+ reported by cases versus controls (*n* = 203)*. *p-Values calculated using Fisher’s exact test.

In the comparison of barriers to retention among cases and controls, lack of time to attend clinic, difficulty with money for transport to clinic, ART side effects, and poor treatment by clinic staff were more commonly reported by cases (Figure 1). There were no significant differences for other barriers, including desire to see a traditional healer or take non-ARV treatments, remembering to take ART every day, fear of individuals finding out about HIV status, lack of partner support, and lack of privacy at health centres.

All women were asked about types of support (facilitators) that would help them to engage in ART as part of the Option B+ programme, and 200 (98.5%) responded affirmatively to at least one facilitator. For the combined cohort of cases and controls, women most frequently requested support for growing food (84.2%), support from primary partner (79.3%), more education about HIV and ART (78.2%), having been HIV tested with the primary partner (75.7%), and support groups either in the community (73.4%) or in clinic (71.9%). Women retained in care were more likely to respond affirmatively to assistance with disclosure despite the fact that all had disclosed to their primary partner. They were also more likely to respond affirmatively to the facilitators of support from primary partner, assistance for disclosure to those outside of primary partnership (family, friends, and community), and more privacy at the health facility (Table 3).

**Table 3.** Summary of facilitators to retention in Option B+ reported by cases and controls (*n* = 200).

Facilitators^a^ (*What types of support would have helped you or would make it easier for you to take ART?)*	Number of women in control group responding yes (%)	Number of women in case group responding yes (%)	*p*-Values^b^
Support for growing food	133 (86.9)	38 (76.0)	0.08
Support from primary partner	128 (83.7)	33 (66.0)	0.01
More education about HIV, ART, and how to stay healthy	122 (80.3)	36 (72.0)	0.23
Having been HIV tested with primary partner	117 (77.0)	36 (72.0)	0.48
Support groups in the community	117 (77.0)	32 (64.0)	0.09
Support groups in clinic	114 (74.5)	32 (64.0)	0.16
Assistance to disclose to primary partner	104 (68.0)	24 (48.0)	0.01
Assistance to disclose to others (family, friends, and community members)	104 (68.0)	23 (46.0)	0.01
Better privacy at the health centre	104 (68.0)	23 (46.0)	0.01
Having an ART clinic closer to your home	91 (59.5)	29 (58.0)	0.85
Decrease in the number of times needed to visit the health centre for ART refills	76 (49.7)	23 (46.9)	0.74
Help remembering to take ART or come to clinic from a person	72 (47.1)	18 (36.0)	0.17
Help remembering to take ART or come to clinic with a phone call or text message	64 (41.8)	16 (32.0)	0.21

^a^Women could respond yes to more than one type of support.

^b^Calculated by chi-square or Fisher’s exact tests.

## Discussion

Data from this study support the published literature demonstrating that younger age [7,9], lower level of education [9], and longer distance from clinic [9] are important barriers to retention in Option B+. Our data add to the existing literature by showing that when controlling for the aforementioned known factors, disclosure to the primary partner and awareness of partner HIV status remains strongly associated with retention. In a cross-sectional study of Option B+ in Ethiopia, disclosure was found to be associated with good adherence to ART [11], and a study from Papua New Guinea found that having a partner tested was associated with retention and infant HIV-free survival [12]. A small qualitative study from Zimbabwe showed benefit of a community-based mentor mother programme on retention, but noted non-disclosure as an important barrier that prevented participation in this type of support programme [13]. Likewise, two qualitative studies of Option B+ women in Malawi found non-disclosure as a reason for stopping ART [14,15]. In both of these studies, starting ART on the same day of HIV diagnosis was a barrier to retention because it resulted in lack of time to accept the diagnosis and did not allow women to disclose and gain partner support for treatment. While our study did not show an association between same-day ART initiation and lower retention, several other studies have reported this finding [7,8,16].

Interventions addressing disclosure could result in significant improvements in Option B+ retention and ideally should include strategies to engage male partners and foster supportive partnerships in order to have sustained benefit. The importance of addressing gender inequality as a means to end vertical transmission of HIV has been raised with a solution of “gender transformative PMTCT programmes” [17]; however, translating policy into practice remains a challenge. For women who feel unable to start ART on the same day of HIV diagnosis due to issues with disclosure and/or securing partner support, allowing a short delay in initiation during which they can be linked to support (assisted disclosure) may facilitate uptake and retention. This should be done in the setting of established tracing protocols, in the event they do not return for care. Treatment delays must be weighed against benefits of treatment during pregnancy, particularly for those diagnosed with HIV late in pregnancy [18].

In addition to disclosure, lack of knowledge about HIV/ART has also been raised as a barrier to retention in other small studies of Option B+ women. In a study from Malawi, 10% of women reported limited understanding of ART as a reason for stopping treatment [9]. Women participating in two qualitative studies in Malawi also reported insufficient understanding of the importance of ART for life and felt they had inadequate counselling about Option B+ prior to initiation [14,15]. It is possible that the move to “Test and Start” in Malawi, in which all HIV-infected individuals will be offered ART, will reduce scepticism around the need for lifelong ART by removing qualifications for ART among subpopulations and through education campaigns targeting the benefits of ART for all HIV-infected individuals. However, pregnant and postpartum women remain uniquely vulnerable to loss to follow-up, and targeted education for this group is important. Women may need information and reassurance about the safety of ART during pregnancy and breastfeeding, as well as the safety of conceiving on ART. Additionally, understanding the benefits of ART for prevention of transmission to HIV-uninfected partners could be a powerful motivator for women to remain in care. Counselling and education interventions should be inexpensive, scalable, and cost-effective.

In our survey of barriers to retention in Option B+, we found that time to travel to clinic, costs of traveling to clinic, and poor treatment by clinic staff were more commonly reported by women who were lost to follow-up. Differentiated models of care focused on Option B+ women may be one important strategy for addressing these barriers. A study from Malawi showed that “models of care” in which women are treated are associated with retention. Investigators found highest retention when ART initiation and maintenance were both done within ART clinics rather than at ANC [19]. A second randomized study from Kenya evaluated integration of ANC and PMTCT services and found that benefits were seen for uptake of ART but not for retention [20]. Strategies that reduce the burden of time and cost for travel to clinic include community ART groups or treatment adherence groups where ART is distributed locally in a supportive setting with other HIV-infected pregnant and postpartum women on ART. These types of models may be particularly facilitative for those experiencing poor treatment in clinical settings, as was reported by women in our population and in other studies of PMTCT [21,22].

Side effects of ART emerged as another important barrier for women lost to follow-up from Option B+. Two other small studies from Malawi have reported this same finding for Option B+ women. In the first study, side effects were reported by 10% of women who stopped ART [9], and in the second study, half of women (13/26) who stopped ART reported stopping due to side effects. In this latter cohort, almost all women reported at least one side effect, largely within the first few weeks of initiation [14]. There are several studies of integrase inhibitor-based ART in development, including one in pregnant and postpartum women [23]. These regimens may result in improved tolerability and are likely to be available in generic, low-cost formulations. Pending results of these clinical trials and the availability of newer regimens, counselling around side effects of ART, and support for women experiencing challenges with tolerability, including options to switch regimens, may help to improve retention in Option B+.

Women identified a number of types of support that would improve their ability to start and remain on ART. Women retained in care were more likely to identify the need for support for disclosure despite the fact that they all reported disclosure to their primary partner. Perhaps, having performed disclosure provided insight into the need for better preparation for this experience. Many of the factors identified by women in our study have been identified in other cross-sectional studies of PMTCT in the pre-Option-B+ era or in the setting of Option B+, including nutrition support for improved food security [14,21,24], support groups [21,25], and community health worker programmes [14,26]. Women reported HIV testing with their partner as a potentially helpful facilitator. In a recent study in Malawi, pregnant women were randomized to receive an invitation for partner HIV co-testing in ANC versus receiving an invitation for co-testing with tracing if the partner did not return for testing [27]. Invitation with partner tracing was found to increase the proportion of couples HIV tested together without causing harm. The impact on retention in care was not assessed and should be investigated in further studies.

There have been few interventional studies on strategies to improve retention. A South African study of mobile-phone-based case management in Option B+ women showed that phone communication was acceptable and improved rates of HIV infant testing but did not have an impact on 12-month retention, although efficacy analysis was limited by small sample size [28]. Thirty-nine per cent of women in our study identified cell phone reminders as a type of support that might help them take ART and return for clinic appointments. However, results from this South African study may not be easily generalized to Malawi due to differences in availability of mobile phones and mobile coverage, particularly in remote rural areas. The Promoting Retention among Infants and Mothers Effectively (PRIME) study is a cluster randomized intervention being performed in Malawi and will evaluate the effectiveness of text message reminders for mother–infant pairs who miss appointments, with the primary outcome of 12-month postpartum retention [29]. A second large randomized study in Malawi, The PMTCT Uptake and Retention (PURE) study, is a three-arm cluster randomized trial to evaluate clinic- or community-based peer support with retention as an outcome for women, infants, and male partners [30]. These studies will provide much needed rigorous data on strategies to improve Option B+ retention.

### Limitations

Our study was limited by the small number of women who defaulted (cases), which limited the number of factors included in the multivariate model. We initially planned to enrol cases in a 1:1 fashion but had difficulty tracing women who had been lost to follow-up. Additionally, women who defaulted and were successfully traced may be different from women who cannot be traced. We performed the study to gain preliminary data for larger interventions and, therefore, did not correct for multiple statistical tests. Our survey was administered by a female Malawian trained in quantitative and qualitative research data collection; however, women in Malawi can be reluctant to report their experiences in a research setting. It is possible that women did not reveal all relevant barriers during the survey. Our sample included women from central Malawi and, therefore, may not be generalizable to all regions in Malawi or other settings in Sub-Saharan Africa.

## Conclusions

Interventions that address partner disclosure and gender dynamics and those that strengthen pre-ART education around the benefits of ART for maternal and child health should be evaluated as strategies to improve retention in Malawi’s Option B+ programme. Differentiated models of care for pregnant and postpartum women hold promise as strategies to help overcome additional barriers to retention, such as time and cost of travel to ART clinics. Consideration should be given to whether ART side effects are playing a role in adherence and retention. The era of Test and Start may result in improved identification of HIV-infected men and raise opportunities for partner testing and family-based support for adherence and retention.
